# Functional Latissimus Dorsi Transfer for Upper-Extremity Reconstruction: A Case Report and Review of the Literature

**Published:** 2017-02-17

**Authors:** Aditya Sood, Paul J. Therattil, Gerardo Russo, Edward S. Lee

**Affiliations:** Division of Plastic and Reconstructive Surgery, Rutgers New Jersey Medical School, Newark

**Keywords:** upper-extremity reconstruction, latissimus dorsi flap, flap reconstruction, functional muscle transfer, elbow flexion

## Abstract

**Objective:** The latissimus dorsi flap is a workhorse for plastic surgeons, being used for many years for soft-tissue coverage of the upper extremity as well as for functional reconstruction to restore motion to the elbow and shoulder. The authors present a case of functional latissimus dorsi transfer for restoration of elbow flexion and review the literature on technique and outcomes. **Methods:** A literature review was performed using MEDLINE and the Cochrane Collaboration Library for primary research articles on functional latissimus dorsi flap transfer. Data related to surgical techniques and outcomes were extracted. **Results:** The literature search yielded 13 relevant studies, with a total of 52 patients who received pedicled, functional latissimus dorsi flaps for upper-extremity reconstruction. The most common etiology requiring reconstruction was closed brachial plexus injury (n = 13). After flap transfer, 98% of patients were able to flex the elbow against gravity and 82.3% were able to flex against resistance. In the presented case, a 77-year-old man underwent resection of myxofibrosarcoma of the upper arm with elbow prosthesis placement and functional latissimus dorsi transfer. The patient was able to actively flex against gravity at 3-month follow-up. **Conclusions:** A review of the literature shows that nearly all patients undergoing functional latissimus dorsi transfer for upper-extremity reconstruction regain at least motion against gravity whereas a large proportion regain motion against resistance. Considerations when planning for functional latissimus dorsi transfer include patient positioning, appropriate tensioning of the muscle, safe inset, polarity, management of other affected upper-extremity joints, and educating patients on the expected outcomes.

## BACKGROUND

Prior to the 1970s, the amputation rate for upper-extremity sarcomas was 40% to 50%.^1^ Rosenberg et al,^2^ however, demonstrated that limb-sparing sarcoma resection with radiation therapy yielded at least equivalent results to limb amputation with regard to 5-year disease-free and overall survival. Their analysis indicated that the only variable that increased local recurrence was positive tumor margins. To minimize the risk of local recurrence, orthopedic and surgical oncologists need to obtain negative margins with extirpative impunity. Aggressive resection needs to be dovetailed with preservation of limb function. Preservation of function may necessitate transfer of muscle groups, preservation of length, soft-tissue reconstruction, and bony reconstruction.

The latissimus dorsi (LD) flap is a workhorse for plastic surgeons, being used for many years for soft-tissue coverage of the upper extremity as well as for functional reconstruction.^3^ Other flaps helpful for upper-arm and elbow defects include local flaps, the pedicled radial forearm flap, reverse lateral arm flap, and free tissue transfer. Although the LD flap may be used solely for soft-tissue coverage, one of the most unique functions of this flap is restoration of upper-extremity function (shoulder abduction, elbow flexion/extension). The flap is able to provide this function with minimal donor site morbidity.^4^ The purpose of this study was to review the literature examining the various techniques of functional LD flap transfer for upper-extremity reconstruction and methods to optimize outcomes.

## METHODS

### Case description

**Figure 1:**
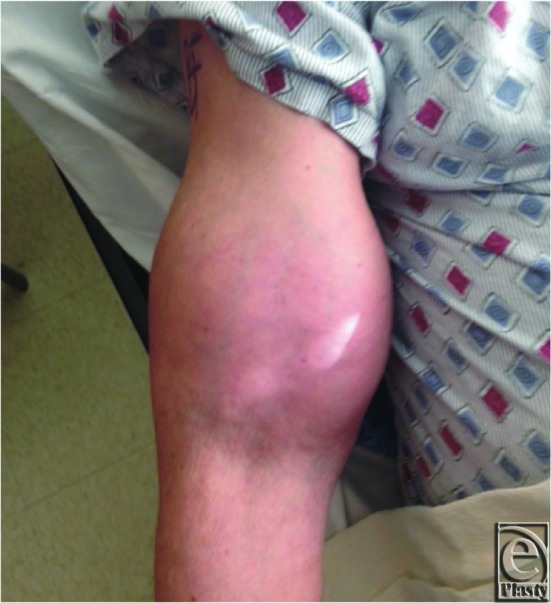
Preoperative view of a slow-growing soft-tissue mass of the right arm.

**Figure 2:**
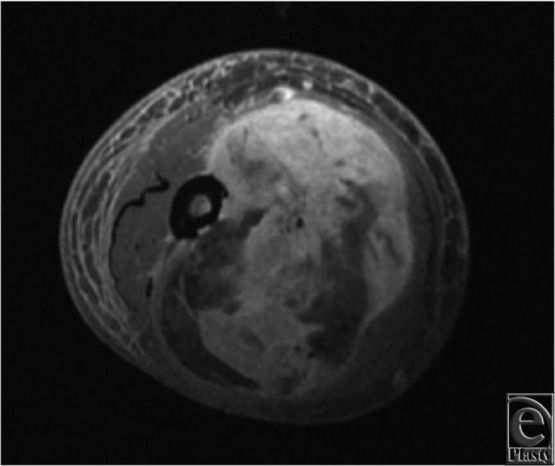
Magnetic resonance imaging of myxofibrosarcoma with invasion into the underlying humerus.

**Figure 3:**
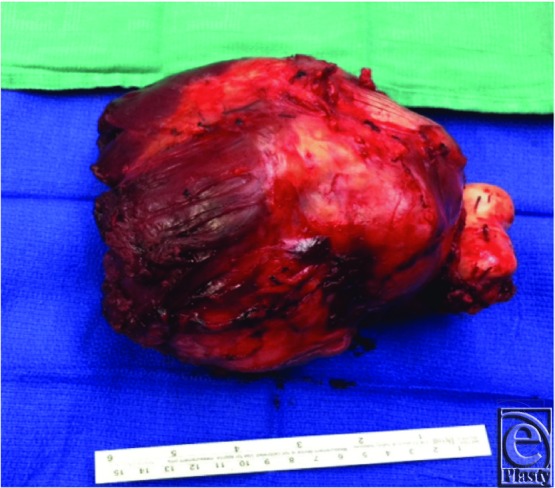
Intraoperative view of the myxofibrosarcoma specimen after resection.

The authors present a case of a pedicled, functional LD flap for reconstruction of a soft-tissue defect after upper-extremity sarcoma extirpation. The patient was a 77-year-old, right-hand–dominant man who initially presented to the orthopedic oncology team with a slow-growing soft-tissue mass (13.9 × 12.0 × 9.0 cm) at the anterior aspect of the right proximal arm (Fig 1). Pathologic analysis from an excisional biopsy indicated myxofibrosarcoma involving muscle and distal humerus. The sarcoma occupied most of the anterior compartment of the arm (Fig 2). Neurovascular structures were intact. After undergoing radical resection of the mass (Fig 3), prosthetic total elbow replacement was performed.

### Literature search

PubMed and Cochrane databases were thoroughly searched by the authors from January 1975 to March 2016. In addition, bibliographies of each relevant citation were reviewed for additional sources. The following search terms were used as both subjects and key words: “latissimus dorsi flap” AND (“upper extremity” OR “elbow” OR “shoulder” OR “arm”). The initial PubMed search yielded 410 studies. The Cochrane database search yielded 2 studies. Two independent reviewers evaluated the titles and abstracts of all studies without language restrictions and subsequently included or excluded studies based on the inclusion and exclusion criteria.

The authors included studies that were published in scientific journals and involved patients who underwent a pedicled, functional LD transfer to the upper extremity. The authors excluded studies that were focused on procedures unrelated to pedicled LD flaps to the upper extremity, including those related to free LD flaps, and those LD flaps that were not specifically intended to restore function. Studies that did not report functional outcomes were also excluded.

Articles of abstracts that met criteria were reviewed as a second stage. Discrepancies between the authors were discussed, and a third senior author made the decision as to whether the study should be included or excluded. The final pool comprised 13 studies with a total of 52 patients (Table 1). Techniques involving functional LD transfer to the upper extremity were evaluated and compared, as were outcomes related to flap transfer.

## RESULTS

### Case description

**Figure 4:**
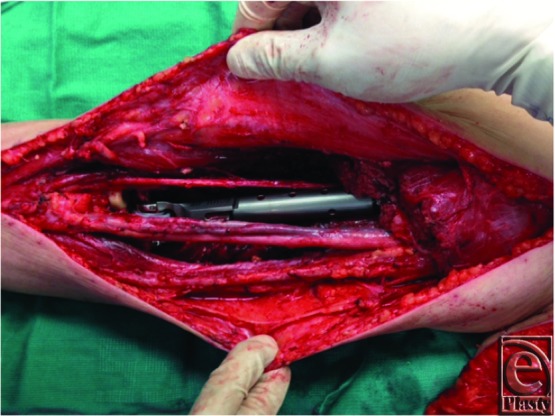
Intraoperative view of the resulting arm defect after tumor extirpation with exposed prosthesis and neurovascular structures.

**Figure 5:**
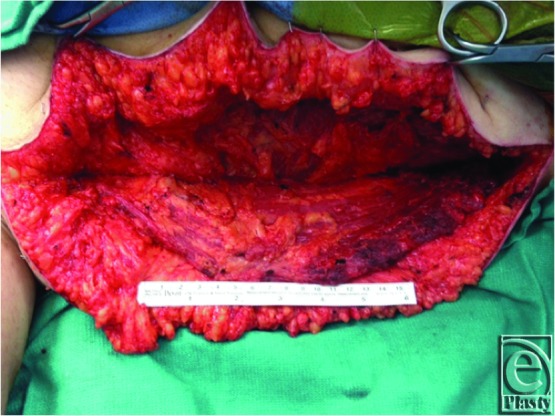
Intraoperative view of dissection of unipolar pedicled latissimus dorsi muscle flap.

The plastic surgery team was requested for coverage of the resulting defect (Fig 4). Functional reconstruction using LD muscle transfer for prosthetic coverage was performed (Fig 5, see Video 1). The flap was sutured to the proximal ulna, periosteum, and joint capsule. At last follow-up, 3 months postoperatively, the patient was able to perform elbow flexion, extension, pronation, and supination. Elbow flexion against gravity was possible (grade M3).

### Literature search

The 13 studies included in our analysis comprised a total of 52 patients who received pedicled, functional LD flaps for upper-extremity reconstruction. Of the 52 patients, etiologies of injury included closed brachial plexus injuries (n = 13), transhumeral amputation for various reasons (n = 12), unspecified trauma (n = 7), poliomyelitis (n = 6), sarcoma (n = 2), Erb's palsy (n = 4), caustic injection (n = 1), animal bite (n = 1), ischemic necrosis (n = 1), humeral fracture with nerve injury (n = 1), burn (n = 1), crush injury (n = 1), nerve laceration (n = 1), and 1 case with etiology not reported.

The delay from time of injury to the time of functional LD flap was not reported in 61.5% (8/13) of studies. In the remainder, the time ranged from 0 to 23 months. There was 1 flap loss in 53 patients (1.9%). Outcomes for all studies were reported on BMRC (British Medical Research Council) grading scale (no contracture = 0; trace contracture = 1; active movement without gravity = 2; active movement against gravity = 3; active movement against resistance = 4; full power = 5). Outcome data were not available for 1 patient. All other patients regained function of at least M3 (98.0%). No patients were graded at M1 (0.0%), 1 patient at M2 (2.0%), 8 patients at M3 (15.7%), 38 patients at M4 (74.5%), and 4 patients at M5 (7.8%). Strength based on kilograms lifted with the affected extremity was reported in 53.8% (7/13) of studies and ranged from 0 to 60 kg. Outcomes for each individual were not provided in all studies and thus means for outcomes could not be calculated.

## DISCUSSION

### LD flap variants for defects of the upper extremity

The LD flap is unique in that it is a distant pedicled flap for arm and elbow defects that does not require staged reconstruction. Such distant pedicled flaps requiring division at a second stage include the thoracoepigastric flap, lateral thoracic flap, external oblique fasciocutaneous flap, pectoralis major flap, and rectus abdominis flap. In addition, the LD flap is of significant size compared with alternative locoregional pedicled options such as the anconeus and radial forearm flap.^17^ The limitation of the LD flap is that reconstruction of defects distal to the olecranon may have higher rates of complication secondary to necrosis of the distal LD flap.^18^

Variants of the pedicled LD flap based on the thoracodorsal system have been shown to be successful for upper-extremity soft-tissue reconstruction. Some described variants are the thoracodorsal artery perforator flap, latissimus dorsi musculocutaneous (LDMC) flap, and LD muscle flap.^19^ Because of its unique proximal pedicle branching, the LD flap can also be performed as a split-latissimus based on the transverse and descending branches. This may be useful in head and neck reconstruction where both skin and lining are needed, or in patients in whom harvest of the full muscle would result in unacceptable weakness.^20^

A rarely used advantage of the LD flap is its potential to include an osseous component. Significant injury to the upper arm can result in humeral damage requiring reconstruction of both the bone and soft tissue. In such cases, a composite rib-LD, or scapula-LD, osteomusculocutaneous flap can be utilized to reconstruct the bony loss, fill the soft-tissue defect, and potentially restore elbow flexion with a single pedicled flap transfer.^21^^,^^22^

### Evolving indications for LD flaps in the upper extremity

The use of various types of LD flaps is well established for reconstruction and resurfacing of soft-tissue defects in the upper extremity, including for defects after tumor extirpation. With advances in the adjuvant treatment of extremity sarcomas, stable soft-tissue coverage is even more critical. Because of the difficulty in obtaining clear margins near critical structures in the upper extremity, in addition to external beam radiation therapy, high-dose brachytherapy has been added to postoperative regimens to decease local recurrence. The risk of local wound complications is thought to be even higher with high-dose brachytherapy and thus pedicled LD flaps to upper-extremity sarcoma defects may decrease the risk of exposure of brachytherapy catheters.^23^

### Functional LD flap transfers

The first descriptions of LD muscle transfer for restoration of upper-extremity function by Schottstaedt et al,^24^ and eventually popularized by Zancolli and Mitre,^10^ were used in patients with poliomyelitis. Since then, the functional LD muscle transfer has become a “workhorse” flap in upper-extremity salvage. The most frequent indications for LD flap transfer for elbow flexion include brachial plexus injury, for deficits after arm replantation, and for destruction of the anterior arm musculature.

In cases of extensive injury to the upper extremity, transhumeral amputation may be necessary but requires soft-tissue coverage to maintain humeral length for prosthesis placement. Kesiktas et al^25^ presented a series of 12 patients with high-voltage electrical injuries to the upper extremity with extensive damage to the elbow flexors. Extensive debridement of the elbow flexors with transhumeral amputation resulted in defects with exposed distal humerus and neurovascular structures requiring coverage with an LD flap. The flaps were sewn to the deltoid to assist in shoulder motion. With augmentation of motion by the LD flap, shoulder extension and abduction were maintained, but flexion, external rotation, and internal rotation were significantly decreased from normal range of motion.^25^

Patients may have deficits after oncologic resection affecting a specific muscle group. Muramatsu et al^26^ presented a series of 5 patients undergoing sarcoma resection of the upper extremity resulting in complete loss of the deltoid. All patients had LD flap transfer for restoration of deltoid function. The flap was completed, raised, and detached except for the pedicle. The insertion was sewn to the deltoid tuberosity, whereas the origin was sewn to the clavicle, acromion, and spine. All patients had greater than 160° of shoulder abduction and excellent function (mean = 92% and range = 87%-93% on Musculoskeletal Tumor Society scores).^26^ This maintenance of function is in line with prior series published on preservation of deltoid function via LD flap transfer.^27^ Similar use of the LD flap has been used in patients with massive rotator cuff tears.^28^

Similarly, patients undergoing sarcoma resection may require restoration of triceps function. Schwab et al^29^ described the use of a pedicled LDMC flap for coverage of prosthetic elbow joints in conjunction with functional triceps restoration. In this series, the LDMC flap was sewn to the distal aspect of the triceps. All 5 patients were able to extend the elbow against gravity.^29^

If the fascial origin of the LD muscle is left intact during harvest, the muscle has an even longer arc or rotation. Doi et al^30^ took advantage of this increased arc and were able to perform pedicled, functional LD muscle transfer to restore finger flexion and extension.

To optimize outcomes with functional LD flap transfer, several tenets should be followed. Before any surgical intervention, the preoperative strength of the muscle should be tested, as the muscle is expected to lose a grade of strength when transferred. The patient should start with both the elbow and the arm flexed at 90° and then should be asked to internally rotate and extend the arm as though they are climbing a ladder. The examiner should resist the motion with one arm while palpating the LD muscle with the other hand.^16^ Zancolli and Mitre^10^ recommend only performing the transfer if the muscle is strong enough to adduct the arm against resistance. There is some debate as to whether electromyography is necessary to determine if there is denervation, especially if the injury is neurologic versus traumatic.^31^ Stern et al^11^ reported success in functional LD muscle transfer regardless of the evidence of denervation on electromyography and thus believed it was unnecessary.

When harvesting the LD flap for upper-extremity transfer, the approach is usually through an incision parallel to the free anterolateral border of the muscle. As with other LDMC flaps, it is sometimes helpful to sew the overlying skin paddle to the muscle to prevent shearing during transposition. As with all pedicled flaps, prevention of kinking of the pedicle is critical, as there have been described failures of the pedicled LD flap secondary to kinking.^13^

The most difficult and critical part of the procedure to perform correctly is likely the inset. Prior to transfer, it is important to determine the resting length of the LD. The resting length can be determined by placing the muscle on stretch after the LD has been mobilized while the origin and insertion are still intact. The LD will be on maximal stretch when the arm is abducted, flexed, and externally rotated. Sutures should be placed every 5 cm or so to mark the resting length. Once transferred to the recipient site, the muscle should be stretched back to its resting length such that the sutures that have been placed are again sitting 5 cm apart. Setting the tension of the transfer LD flap is important and should generally be done with the elbow in extension. This will ensure that the elbow will be able to complete full extension.^31^^,^^32^ Some authors prefer to place the elbow in 90° to 100° of flexion and in full supination in order to determine the appropriate resting length.^10^^,^^33^

Functional transfer of the LD flap may be unipolar or bipolar. The choice between unipolar and bipolar transfer of the LD flap is mainly determined by surgeon preference. In the small case series that differentiated between the 2 types of transfer, there was no difference seen in outcomes.^16^ When performing bipolar transfer, the insertion is usually relocated to coracoid process and the origin to the proximal forearm.^6^ Whether it is best to relocate the origin or insertion of the LD flap first is unclear, and each surgeon seems to have his or her own preference.^10^^,^^31^ Cambon-Binder et al^6^ presented a series of 7 patients who underwent functional LD flap transfer for elbow flexion paralysis. In most cases, the insertion was relocated to the coracoid process. The origin was detached and relocated either to the radial tuberosity and the proximal third of the ulna or to the biceps. Patients were immobilized for 6 weeks before physical therapy was started.^6^ If attaching the functional flap transfer to the biceps, the LD muscle can be weaved into the remaining biceps tendon. If attaching the flap to radius, bone anchors may be used or holes may be drilled through the bicipital tuberosity to tie over. It is important to prepare the bone with a high-speed burr or curettes when attaching the muscle flap to radius.^33^

There has been debate with regard to fusion of the glenohumeral joint when performing functional muscle transfers in patients with preexisting brachial plexus injury. Arthrodesis has been suggested by some because of the instability of the shoulder girdle in this population. However, because the LD muscle transfer runs anterior to the glenohumeral joint, it may act as a stabilizer and may preclude arthrodesis of the shoulder.^13^

### Recent advances in LD flap techniques

Several authors have described techniques to improve the continuous challenges around coverage of wounds at the upper extremity using variations of the LD flap. Nazerani et al^34^ presented an approach that addressed the challenge of providing adequate coverage of very large circumferential defects of the upper extremity. By placing a tissue expander in a longitudinal direction and deep to the LD, the flap was expanded to create a longer flap. The flap was then wrapped into the defect in a spiral fashion. Defects from burn scars, large hairy nevi, and traumatic injuries to the arm involving up to 7% body surface area were successfully closed using this technique. These cases suggest that longitudinal tissue expansion of the LD flap with wrapping is safe and effective for treatment of large circumferential defects using a single flap.^34^

Wong and Saint-Cyr^35^ presented an LD flap technique to minimize the loss of LD muscle function. In their series, 5 patients underwent reconstruction and resurfacing with a pedicled, muscle-sparing LDMC flap for treatment of upper-extremity soft-tissue defects. The flap was based on the descending branch of the thoracodorsal artery. The authors also reported that orienting the skin paddle transversely rather than vertically yielded several supposed benefits, including minimal seroma formation, superior aesthetic outcome at the donor site, and more versatility in harvesting larger skin paddles with wider arcs of rotation at 2 axes.^35^

With regard to the flap harvest itself, Boa et al^36^ describe using a dorsal decubitus position versus a traditional lateral decubitus position. This position enables 2 surgeons to operate at the same time when dealing with an upper-extremity defect, thus reducing operative time since repositioning the patient is no longer necessary.

### Functional outcomes with LD flap transfer

Revisions may be needed in a proportion of patients undergoing functional LD flap transfer for restoration of elbow flexion. In the series of 10 patients by Kawamura et al,^16^ 50% did not achieve sufficient elbow flexion after initial LD transfer. The muscle was deemed to be too long and thus was shortened at the distal end of the transfer. Ultimately, 80% of patients acquired strong elbow flexion in this series.^16^ Those patients who did not achieve the intended goal had preoperative weakness of the LD. As can be seen from the aforementioned literature review, almost all patients undergoing functional LD transfer for upper-extremity reconstructions regain at least motion against gravity whereas a large proportion regain motion against resistance.

With regard to donor site morbidity, a systematic review by Lee and Mun^37^ revealed minimal dysfunction after LD muscle flap harvest. Functional morbidity was evaluated with patient questionnaire, DASH (disabilities of the arm, shoulder, and hand) scores, and shoulder range of motion and strength. Forty-one percent of patients experienced some degree of discomfort at the donor site. While there was only mild dysfunction, particularly in the early postoperative period, and DASH scores showed little difficulty in activities of daily living, some studies did demonstrate shoulder weakness over time and difficulty with sports and art activities.^37^

Alternatives to the pedicled, functional LD flap for restoration of elbow flexion are the Steindler flexorplasty, anterior transposition of the triceps, pectoralis major transfer, sternocleidomastoid transfer, and free flap transfer. Flexion contractures of the elbow and wrist are frequent complications of the Steindler flexorplasty. The rate of elbow flexion contracture is thought to be less with pedicled LD muscle transfer, reported at 10% to 30%.^11^^,^^16^^,^^38^^-^40 Issues with transfer of the pectoralis major include diminished strength and excursion of the muscle over time, prominent scar, and excessive adduction of the arm.^41^

## CONCLUSIONS

The LD flap is versatile and can be used for soft-tissue coverage as well as restoration of movement of the upper extremity. The LD flap has minimal donor site morbidity. As can be seen from the aforementioned review of the literature, almost all patients undergoing functional LD transfer for upper-extremity reconstruction regain at least motion against gravity whereas a large proportion regain motion against resistance. Considerations when planning for functional LD transfer include preoperative muscle strength evaluation, patient positioning, appropriate tensioning of the muscle, safe inset, polarity, management of other affected upper-extremity joints, and educating patients on the expected outcomes.

## Figures and Tables

**Table 1:** Characteristics of case series presenting pedicled, functional latissimus dorsi flap transfer

Authors	Years	Number of patients	Patient age, y	Etiology	Surgical delay (average months)	Follow-up (average months)	ROM (average degrees)	Function (BMRC grading scale)	DASH score	MSTS score	Strength (average kg lifted)	Major complications
Cambon-Binder et al^6^	2003-2009	7	18-49	Tiger bite (n = 1), MVC (n = 2), ischemic necrosis (n = 1), brachial plexus palsy (n = 3)	19 (range, 6-48)	26.6 (range, 13-48)	91 (range, 45-130)	M4 (n = 5), M3 (n = 1), M2 (n = 1)	NR	NR	2.14 (range, 0-8)	
Grinsell et al^5^	2006-2010	1	NR	Sarcoma (n = 1)	0	15	NR	M4	31	25	NR	
Lazar et al^7^	2009	1	41	Caustic injection (n = 1)	NR	60	NR	M5	NR	NR	NR	
Schoeller et al^8^	1997-2002	5	7-55 (mean = 35.5)	Upper-arm amputation (n = 5)	(range, 0.5-12)	43 (range, 22-65)	NR	M4 (n = 3), M3 (n = 2)	NR	NR	NR	
Kawamura et al^16^	1986-1999	10	5-24 (mean = 17)	Brachial plexus injury from MVC (n = 8), Erb's palsy (n = 1), humeral fracture with nerve injury (n = 1)	23 (range, 10-61)	55.6 (range, 10-114)	111 (range, 60-140)	M4 (n = 8), M3 (n = 2)	NR	NR	NR	
O'Ceallaigh et al^9^	1998	1	35	Burn (n = 1)	NR	9	100	M4	NR	NR	4	
Zancolli and Mitre^10^	1973	8	NR	Poliomyelitis (n = 6), brachial plexus injury (n = 2)	NR	45.9 (range, 13-72)	125 (range, 105-140)	M4 (n = 8)	NR	NR	(range, 0.7-5)	
Bostwick et al^3^	1979	1	NR	Laceration of musculocutaneous nerve (n = 1)	NR	18	NR	M5	NR	NR	60	
Stern et al^11^	1982	2	12-25 (mean = 18.5)	Upper-arm amputation (n = 2)	NR	4.5 (range, 4-5)	132 (range, 125-140)	M4 (n = 2)	NR	NR	4.5	
Brones et al^12^	1979	1	19	Crush injury (n = 1)	NR	14	NR	M4	NR	NR	6.8	
Stern and Carey^13^	1979-1985	10	3-37 (mean = 19)	Erb's palsy (n = 3), MVC (n = 3), trauma (n = 3), sarcoma (n = 1)	NR	37.6	125 (range, 90-155)	M4 (n = 6), M3 (n = 3)	NR	NR	NR	Failed flap (n = 1)
Haas et al^14^	2002	2	19-21 (mean = 20)	Upper-arm amputation (n = 2)	0	23 (range, 10-36)	NR	M4 (n = 2)	NR	NR	4 (range, 3-5)	
Parmaksizoglu and Beyzadeoglu^15^	1991-2000	3	15-25 (mean = 20.6)	Upper-arm amputation (n = 3)	NR	68 (range, 14-121)	90 (range, 80-100)	M5 (n = 2), M4 (n = 1)	NR	NR	NR	

ROM indicates range of motion; BMRC, British Medical Research Council; DASH, disabilities of the arm, shoulder, and hand; MSTS, Musculoskeletal Tumor Society; MVC, motor vehicle collision; and NR, not reported.

## References

[1] Abbas JS, Holyoke ED, Moore R, Karakousis CP (1981). The surgical treatment and outcome of soft-tissue sarcoma. Arch Surg.

[2] Rosenberg SA, Tepper J, Glatstein E (1982). The treatment of soft-tissue sarcomas of the extremities: prospective randomized evaluations of (1) limb-sparing surgery plus radiation therapy compared with amputation and (2) the role of adjuvant chemotherapy. Ann Surg.

[3] Bostwick J III, Nahai F, Wallace J G, Vasconez LO (1979). Sixty latissimus dorsi flaps. Plast Reconstr Surg.

[4] Legre R, Boghossian V, Servant JM, Magalon G, Bureau H (1990). [Analysis of sequelae of the latissimus dorsi flap removal. Report of 44 cases reviewed and tested]. Ann Chir Plast Esthet.

[5] Grinsell D, Di Bella C, Choong PF (2012). Functional reconstruction of sarcoma defects utilising innervated free flaps. Sarcoma.

[6] Cambon-Binder A, Belkheyar Z, Durand S, Rantissi M, Oberlin C (2012). Elbow flexion restoration using pedicled latissimus dorsi transfer in seven cases. Chir Main.

[7] Lazar CC, Revol M, Servant JM (2009). Functional and aesthetic outcome of a complex upper-limb reconstruction after criminal caustic injection. J Reconstr Microsurg.

[8] Schoeller T, Wechselberger G, Hussl H, Huemer GM (2007). Functional transposition of the latissimus dorsi muscle for biceps reconstruction after upper arm replantation. J Plast Reconstr Aesthet Surg.

[9] O'Ceallaigh S, Mehboob Ali KS, O'Connor TP (2005). Functional latissimus dorsi muscle transfer to restore elbow flexion in extensive electrical burns. Burns.

[10] Zancolli E, Mitre H (1973). Latissimus dorsi transfer to restore elbow flexion. An appraisal of eight cases. J Bone Joint Surg Am.

[11] Stern PJ, Neale HW, Gregory RO, Kreilein JG (1982). Latissimus dorsi musculocutaneous flap for elbow flexion. J Hand Surg Am.

[12] Brones MF, Wheeler ES, Lesavoy MA (1982). Restoration of elbow flexion and arm contour with the latissimus dorsi myocutaneous flap. Plast Reconstr Surg.

[13] Stern PJ, Carey JP (1988). The latissimus dorsi flap for reconstruction of the brachium and shoulder. J Bone Joint Surg Am.

[14] Haas F, Hubmer M, Koch H, Scharnagl E (2004). Immediate functional transfer of the latissimus dorsi myocutaneous island flap for reestablishment of elbow flexion in upper arm replantation: two clinical cases. J Trauma.

[15] Parmaksizoglu F, Beyzadeoglu T (2003). Functional latissimus dorsi island pedicle musculocutaneous flap to restore elbow flexion in replantation or revascularisation of above-elbow amputations. Handchir Mikrochir Plast Chir.

[16] Kawamura K, Yajima H, Tomita Y, Kobata Y, Shigematsu K, Takakura Y (2007). Restoration of elbow function with pedicled latissimus dorsi myocutaneous flap transfer. J Shoulder Elbow Surg.

[17] Jensen M, Moran SL (2008). Soft tissue coverage of the elbow: a reconstructive algorithm. Orthop Clin North Am.

[18] Choudry UH, Moran SL, Li S, Khan S (2007). Soft-tissue coverage of the elbow: an outcome analysis and reconstructive algorithm. Plast Reconstr Surg.

[19] Tan O, Atik B, Ergen D (2007). Versatile use of the pedicled latissimus dorsi flap as a salvage procedure in reconstruction of complex injuries of the upper extremity. Ann Plast Surg.

[20] Tobin GR, Moberg AW, DuBou RH, Weiner LJ, Bland KI (1981). The split latissimus dorsi myocutaneous flap. Ann Plast Surg.

[21] Unlu RE, Kargi AE, Celebioglu S, Erdoğan B, Sensöz O (2002). Reconstruction of the upper extremity with a compound rib-latissimus dorsi osteomusculocutaneous flap. Scand J Plast Reconstr Surg Hand Surg.

[22] Seghrouchni H, Martin D, Pistre V, Baudet J (2003). [Composite scapular flap for reconstruction of complex humeral tissue loss: a case report]. Rev Chir Orthop Reparatrice Appar Mot.

[23] Lane JD, Pomahac B, Raut CP, Baldini EH, Devlin PM (2014). High-dose-rate interstitial brachytherapy boost with a pedicled latissimus dorsi myocutaneous flap for myxofibrosarcoma of the arm. Plast Reconstr Surg Glob Open.

[24] Schottstaedt ER, Larsen LJ, Bost FC (1955). Complete muscle transposition. J Bone Joint Surg Am.

[25] Kesiktas E, Eser C, Gencel E, Aslaner EE, Yavuz M (2015). Reconstruction of transhumeral amputation stumps with ipsilateral pedicled latissimus dorsi myocutaneous flap in high voltage electrical burns. Burns.

[26] Muramatsu K, Ihara K, Tominaga Y, Hashimoto T, Taguchi T (2014). Functional reconstruction of the deltoid muscle following complete resection of musculoskeletal sarcoma. J Plast Reconstr Aesthet Surg.

[27] Mimata Y, Nishida J, Gotoh M, Akasaka T, Shimamura T (2013). Limb function after excision of a deltoid muscle sarcoma. J Shoulder Elbow Surg.

[28] Hart R, Barta R, Nahlik D (2010). [Latissimus dorsi transfer for the treatment of irreparable craniodorsal tears of the rotator cuff]. Acta Chir Orthop Traumatol Cech.

[29] Schwab JH, Healey JH, Athanasian EA (2008). Wide en bloc extra-articular excision of the elbow for sarcoma with complex reconstruction. J Bone Joint Surg Br.

[30] Doi K, Ihara K, Sakamoto T, Kawai S (1985). Functional latissimus dorsi island pedicle musculocutaneous flap to restore finger function. J Hand Surg Am.

[31] Pierce TD, Tomaino MM (2000). Use of the pedicled latissimus muscle flap for upper-extremity reconstruction. J Am Acad Orthop Surg.

[32] Manktelow RT, Zuker RM, McKee NH (1984). Functioning free muscle transplantation. J Hand Surg Am.

[33] Pirela-Cruz MA, Reddy KK, Higgs M (2007). Soft tissue coverage of the elbow in a developing country. Tech Hand Up Extrem Surg.

[34] Nazerani S, Motamedi MH, Keramati MR, Nazerani T (2010). Upper extremity resurfacing via an expanded latissimus dorsi musculocutaneous flap for large circumferential defects: the “spiral” reconstruction technique. Strategies Trauma Limb Reconstr.

[35] Wong C, Saint-Cyr M (2010). The pedicled descending branch muscle-sparing latissimus dorsi flap for trunk and upper extremity reconstruction. J Plast Reconstr Aesthet Surg.

[36] Boa O, Servant JM, Revol M (2011). Dorsal decubitus positioning: a novel method to harvest the latissimus dorsi flap for massive upper extremity defect reconstruction. Tech Hand Up Extrem Surg.

[37] Lee KT, Mun GH (2014). A systematic review of functional donor-site morbidity after latissimus dorsi muscle transfer. Plast Reconstr Surg.

[38] Chuang DC, Epstein MD, Yeh MC, Wei FC (1993). Functional restoration of elbow flexion in brachial plexus injuries: results in 167 patients (excluding obstetric brachial plexus injury). J Hand Surg Am.

[39] Moneim MS, Omer GE (1986). Latissimus dorsi muscle transfer for restoration of elbow flexion after brachial plexus disruption. J Hand Surg Am.

[40] Hirayama T, Tada H, Katsuki M, Yoshida E (1994). The pedicle latissimus dorsi transfer for reconstruction of the plexus brachialis and brachium. Clin Orthop Relat Res.

[41] Wahegaonkar AL, Doi K, Hattori Y, Addosooki AI (2008). Surgical technique of pedicled bipolar pectoralis major transfer for reconstruction of elbow flexion in brachial plexus palsy. Tech Hand Up Extrem Surg.

